# Soluble chemokine (C-X-C motif) ligand 16 (CXCL16) in urine as a novel biomarker candidate to identify high grade and muscle invasive urothelial carcinomas

**DOI:** 10.18632/oncotarget.20737

**Published:** 2017-09-08

**Authors:** Kerstin Lang, Nadine Bonberg, Sibylle Robens, Thomas Behrens, Jan Hovanec, Thomas Deix, Katharina Braun, Florian Roghmann, Joachim Noldus, Volker Harth, Karl-Heinz Jöckel, Raimund Erbel, Yu Chun Tam, Andrea Tannapfel, Heiko Udo Käfferlein, Thomas Brüning

**Affiliations:** ^1^ Institute for Prevention and Occupational Medicine of the German Social Accident Insurances, Ruhr-University Bochum (IPA), Bochum, Germany; ^2^ Department of Urology, Universitätsklinik Marien Hospital Herne, Ruhr University Bochum, Herne, Germany; ^3^ Institute of Occupational and Maritime Medicine, University Clinic Hamburg-Eppendorf, Hamburg, Germany; ^4^ Institute for Medical Informatics, Biometry und Epidemiology (IMIBE), University Hospital of Essen, Essen, Germany; ^5^ Department of Cardiology, West-German Heart Center Essen, University Hospital of Essen, Essen, Germany; ^6^ Institute of Pathology, Georgius Agricola Stiftung Ruhr, Ruhr University Bochum, Bochum, Germany

**Keywords:** urothelial cancer, chemokine CXCL16, biomarker, urine, tumour biology

## Abstract

Information on biomarkers of urothelial carcinomas (UC) for clinical decision-making is limited. Here, we newly identified and verified CXCL16 as a promising novel biomarker in urine for high grade and muscle invasive UC in a cross-sectional cohort of 308 UC patients, 126 urological hospital controls, and 50 population controls using antibody arrays and ELISA. Median CXCL16 levels in urine was significantly higher in UC patients (273.2 pg/mg creatinine) compared to hospital (148.1 pg/mg) and population controls (85.1 pg/mg) with a particular preference for high grade (460.8 pg/mg), muscle invasive (535.7 pg/mg) and primary UC (327.8 pg/mg) (all p<0.0001). Group differences were confirmed after adjusting or stratifying for potential clinical and individual characteristics, such as leukocyte counts, haematuria, age, gender, and smoking status. In contrast, CXCL16 showed less discriminating power in low grade (244.3 pg/mg), non-muscle invasive (≤pT1, 251.2 pg/mg) and recurrent UC (203.9 pg/mg). In agreement with its function in immune defence, expression of CXCL16 in tissue samples of primary UC patients (n=53) showed only a weak or no immunoreactivity compared to urological hospital controls (n=32). Expression of CXCR6, the G-protein-coupled receptor of CXCL16, remained unchanged. Our findings suggest that evading the immune defence by shedding cell-surface CXCL16 and its increased elimination in urine is a molecular feature of high grade and muscle invasive UC. Therefore, urinary CXCL16 may serve as a useful, simple and non-invasive tool to identify high-risk UC with increased risk of progression at the molecular level.

## INTRODUCTION

Urothelial carcinoma (UC) is one of the most prevalent types of cancer worldwide, with an estimated 386,000 primary (new) cancer cases diagnosed each year. It is considerably more common in males than in females [1], and at the time of initial diagnosis, approximately 80% of patients present with non-muscle invasive cancer (i.e., pTa and pTis) [2]. After treatment however, up to 80% of all cases recur, with approximately 5-10% becoming invasive (>pT1).

Non-invasive biomarkers of UC in urine are of particular importance due to the direct contact of the matrix (urine) with the urothelium. However, information on UC biomarkers for clinical decision-making is limited, although there are several situations where both physicians and patients may benefit from using biomarkers. For example, a urinary biomarker can be a fast and non-invasive diagnostic tool in uncertain situations, such as when patients at risk present with microhaematuria. The patient and the physician can then make an informed decision, i.e., prior to using an invasive technique, such as cystoscopy which can be uncomfortable, especially in male patients. Ideally, such a biomarker should be able to predict any type of UC (general diagnostics). In addition, the presence or absence of a biomarker in urine can also shed some light on specific histological and molecular characteristics of UC, such as grade, invasiveness or molecular type of UC only (companion diagnostics). The results can then be used by the pathologist to confirm the histological evaluation at the molecular level, and by the physician and patient to help plan the most appropriate treatment.

Currently, there are only two FDA-approved protein biomarkers available for non-invasive UC diagnostics in urine - the nuclear matrix protein 22 (NMP22, Bladder Chek^®^) and a variant of human complement factor H (CFHrp, BTA *stat*^®^, BTA TRAK^®^). Both are general diagnostic markers that have been approved for the detection of primary and recurrent UC [3, 4, 5, 6]. Previous cross-sectional studies have reported values of 47-100% for sensitivity and 60-90% for specificity of NMP22 [4, 7, 8], whereas 30-80% sensitivity and 50-90% specificity were reported for CFHrp-associated tests [5, 9]. Although initial studies were promising, the diagnostic value of NMP22 and CFHrp in clinical practice and prospective studies was poor, with high false-positive rates (low specificity) due to strong confounding effects of inflammation and haematuria [10, 11, 12]. The latter two are not only concomitant conditions of UC but, more importantly, characteristics of other urological conditions, which makes the use of NMP22 and CFHrp as general diagnostic markers of UC critical. In fact, testing for NMP22 using BladderChek^®^ is not recommended if leukocytes were positively detected in urine, a clinical sign of urinary tract infection [13]. In such cases, patients are often treated with antibiotics, before being examined once more. In addition to being unfavourable for UC diagnosis in general, NMP22 and CFHrp are also not promising as therapeutic targets or are indicative of particular molecular features of UC. Therefore, the use of NMP22 and CFHrp remains ineffective in companion diagnostics (i.e., guiding therapeutic decisions).

Here, we present data on the soluble chemokine (C-X-C motif) ligand 16 (CXCL16) as a novel biomarker candidate in urine for UC. Based on the above-described potential applications of biomarkers in the clinical decision-making process, we evaluated the diagnostic properties of CXCL16 by comparing CXCL16 in urine of three groups of individuals (UC patients, urological hospital controls without UC, and healthy individuals from the general population) while controlling for individual risk factors (i.e. age, gender, smoking status, patients’ history of UC) and clinical modifying factors (i.e., leucocyte and erythrocyte counts in urine, staging, grading). Finally, we investigated the expression of CXCL16 and its receptor C-X-C chemokine receptor type 6 (CXCR6), a G-protein-coupled receptor with potential therapeutic relevance, in a subset of tissue samples derived from patients with UC compared to hospital controls (i.e., those with urocystitis but no cancer) to gain first insights into a possible role of CXCL16 in UC carcinogenesis.

## RESULTS

### Study population

In the initial antibody array (screening approach), urine samples from six low grade primary UC patients and six hospital controls with pathologically confirmed urocystitis were used (see section Materials and Methods for detail). The initial screen for potential biomarker candidates was carried out semi-quantitatively using a commercially available antibody array capable of simultaneously screening the relative levels of 55 angiogenesis-related proteins. The results showed an approximately 3-fold increase in CXCL16 levels in patients with UC compared to those with urocystitis only. In addition, we found higher urinary concentrations of dipeptidyl peptidase IV (CD26, 3.5 fold), human platelet factor 4 (CXCL4; 8.5 fold) and vascular endothelial growth factor (VEGF; 3.5 fold) in UC patients. However, after further analysis using more specific ELISAs, CXCL16 was the most promising candidate for further evaluation.

Accordingly, CXCL16 results were quantitatively confirmed in spot urine samples from 434 urological inpatients. Urine samples were collected from these patients prior to cystoscopy and upper urinary tract inspection, and treatment by transurethral resection of cancer-suspicious lesions. Subsequent pathological examination of all tissue samples by two pathologists revealed 308 patients with UC (253 men and 55 women; 200 primary UC and 108 recurrent UC); whereas 126 patients (91 men, 35 women) were cancer-free, but histologically diagnosed with urocystitis. These patients were used as urological hospital controls. In addition, 50 healthy subjects without former UC, (39 men and 11 women) were enrolled from the same residential areas (Table 1). Their urine samples were collected and prepared for analysis using identical Standard Operating Procedures (SOPs) done for all hospital inpatients.

**Table 1:** Comparison of normalized CXCL16 values (pg/mg creatinine) according to patient and sample characteristics

	Population Controls (N=50)		Urological Hospital Controls (N=126)		Urothelial Carcinoma (N=308)	
	n	Median (IQR)	n	Median (IQR)	n	Median (IQR)
**Total**	50	85.1 (47.5-136.6)	126	148.1 (87.0-264.8)	308	273.2 (152.8–543.3)
**Creatinine [mg/ml]**	50	0.6 (0.3-1.0)	126	0.8 (0.4-1.2)	303	0.9 (0.6-1.3)
**Age [years]**						
<70	30	81.2 (36.0-131.9)	68	135.2 (84.8-219.6)	121	225.9 (132.1-420.1)
≥70	20	102.0 (57.6-141.0)	58	189.8 (88.6-335.1)	187	284.8 (170.1-597.9)
**Gender**						
Males	39	79.6 (42.5-126.0)	91	146.7 (84.4-274.8)	253	256.7 (149.2–509.5)
Females	11	131.5 (54.2-162.8)	35	160.9 (98.6-255.3)	55	302.1 (195.6-818.3)
**Smoking Status**						
Never smoker	19	91.6 (42.5-162.9)	25	149.9 (109.2-309.4)	55	285.1 (193.6-593.5)
Former smoker	24	80.9 (40.6-112.8)	58	120.9 (69.7-229.0)	146	248 (131.3-504.5)
Current smoker	7	131.9 (53.8-167.2)	32	181.5 (121.4-272.8)	82	295.8 (192.8–545.5)
**Leucocytes**						
Negative	38	80.9 (42.5-122.8)	62	127.1 (75.6-250.2)	160	212.7 (128.8–393.0)
Positive	10	135.6 (78.8-160.3)	61	160.9 (119.1-309.4)	142	352.8 (225.9-582.2)
**Erythrocytes^a^**						
Negative-∼10	46	82.5 (47.1-131.9)	76	123.5 (68.4-197.6)	126	154.3 (101.3-249.7)
∼25-50	2	134.2 (122.8-145.5)	21	184.7 (119.1-290.9)	58	275.4 (203.9-491.5)
∼150-250	0		26	254.0 (195.0-375.1)	119	453.7 (295.1-751.6)
**UC History**						
No	49	87.4 (47.5-136.6)	55	145.6 (98.6-255.3)	199	327.8 (197.8-597.9)
Yes	0		70	149.1 (85.3-274.8)	109	203.9 (129.0-322.4)
**Tumour grading**						
Low grade with UC history					97	193.6 (123.8-285.1)
Low grade without UC history					142	283.6 (141.6-545.5)
High-grade with UC history					12	338.8 (208.8-747.8)
High-grade without UC history					50	515.6 (277.0-835.5)
**Tumour staging^b^**						
pTa					158	194.6 (122.2-306.7)
pT1					73	335.6 (222.7-645.6)
pT2, pT2a, pT2b					7	534.3 (359.4-689.7)

n, number of samples; IQR, Interquartile range; ^a^erythrocytes are displayed in categories of approximate numbers of erythrocytes/μl urine based on Combur-Test^®^ sticks, ^b^low grade tumours only to avoid confounding by high-grade.

### CXCL16 in urine

**Figure 1:**
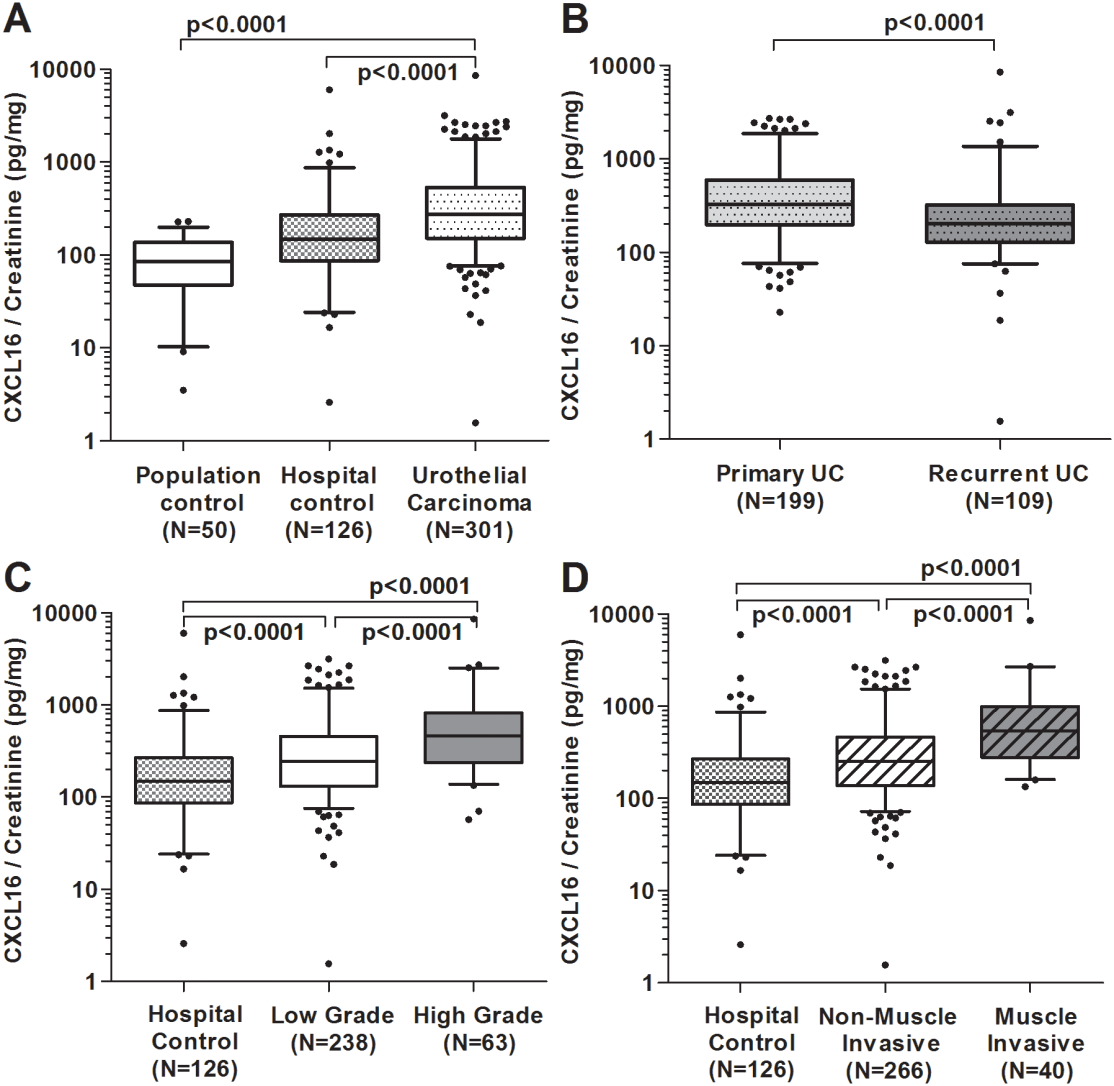
Concentration of soluble CXCL16 measured in urine of patients with urothelial carcinoma (UC) and in hospital or population controls Box plots of creatinine-normalized CXCL16 concentration in urine of all participants. CXCL16 concentration in urine of UC patients compared to population and hospital controls **(A)**. CXCL16 in urine of primary UC patients compared to recurrent UC patients **(B)**. Urinary CXCL16 levels in hospital controls versus low-grade and high-grade UC patients **(C)**. Comparison of muscle invasive (>pT1) with non-muscle invasive (≤pT1) UC patients **(D)**. Non-parametric Wilcoxon rank sum test were performed to examine differences between the groups.

ELISA quantification of CXCL16 in all urine samples confirmed higher levels in patients with UC (median 273.2 pg/mg creatinine) compared to both hospital controls (median 148.1 pg/mg, p<0.0001) and population controls (median 85.1 pg/mg creatinine, p<0.0001; Figure 1A, Table 1). Overall, we observed the anticipated increase in CXCL16 levels in urine with population controls < hospital controls < UC patients. In addition, differences between UC patients and hospital controls were statistically significant for patients with low grade (median 244.3 pg/mg, p<0.0001) and high grade UC (median 460.8 pg/mg creatinine, p<0.0001; Table 1; Figure 1C). Furthermore, median CXCL16 values in muscle-invasive UC patients were significantly higher (535.7 pg/mg) compared to those with non-muscle invasive UC patients (251.2 pg/mg) and hospital controls (148.1 pg/mg, all p<0.0001; Figure 1D). Within the group of UC patients without UC history (=primary UC), we observed higher median concentrations of CXCL16 (327.8 pg/mg creatinine) in comparison to those with former UC (203.9 pg/mg, Table 1; Figure 1B).

To identify the influence of individual characteristics (e.g., age, gender, etc.) on CXCL16 within the study groups, a linear multiple regression analysis was performed (Table 2). Basic factors such as age, gender, and smoking status had no impact on urinary CXCL16 concentration. In addition, we studied the influence of clinically relevant characteristics (e.g., stage, grade, primary and recurrent UC, etc.) on CXCL16 levels in all study subjects. We observed significantly higher CXCL16 values in non-muscle invasive (≤pT1) low-grade (Δ=0.95, 95% CI 0.59 −1.31) and non-muscle invasive high-grade carcinoma patients (Δ =1.09, 95% CI 0.56 – 1.62) compared to the population controls (p≤0.001, Table 2). However, even higher CXCL16 values were determined in muscle-invasive (>pT1) low grade (Δ =1.14, 95% CI 0.17 – 2.12) and muscle-invasive high grade (Δ =1.45, 95% CI 0.92 – 1.99) UC patients. In contrast, the differences to the hospital controls were less pronounced, although still significantly different (Δ =1.03, 95% CI 0.58 – 1.49 for muscle-invasive high grade UC, p≤0.001). The results did not show an influencing factor of urinary leucocytes on CXCL16 in urine. Rather, erythrocytes in urine (more than 10 erythrocytes/μl urine) appeared to significantly increase CXCL16 concentration and having the highest impact when urinary erythrocyte levels were >150 Ery/μl (Δ=0.81, 95% CI 0.54 – 1.07).

**Table 2:** Influence of study group (population controls, hospital controls, low-grade UC patients, high-grade UC patients) and group characteristics on log(CXCL16/Creatinine) determined via multiple linear regression analysis

		N	All Subjects (n=429)			Men (n=341)				Women (n=88)			
			Δ	95% CI	p-value	N	Δ	95% CI	p-value	N	Δ	95% CI	p-value
Intercept			4.16	3.79; 4.53	<0.001		4.08	3.67; 4.50	<0.001		4.90	4.21; 5.59	<0.001
Study group	Population controls		Ref.			37	Ref.			10	Ref.		
	Hospital controls	111	0.42	0.03; 0.81	0.033	81	0.53	0.06; 0.99	0.026	30	0.11	−0.56; 0.78	0.736
	Non muscle invasive (≤pT1) and low-grade UC	211	0.95	0.59; 1.31	<0.001	174	1.01	0.59; 1.44	<0.001	37	0.63	−0.04; 1.29	0.066
	Non muscle invasive (≤pT1) and high-grade UC	27	1.09	0.56; 1.62	<0.001	21	0.94	0.33; 1.56	0.003	6	1.88	0.86; 2.89	<0.001
	Muscle invasive (>pT1), low-grade UC	5	1.14	0.17; 2.12	0.021	4	1.10	−0.01; 2.22	0.053	1	1.34	−0.54; 3.21	0.160
	Muscle invasive (>pT1), high-grade UC	28	1.45	0.92; 1.99	<0.001	24	1.23	0.62; 1.84	<0.001	4	2.83	1.72; 3.93	<0.001
Age (years)	< 70	198	Ref.			154	Ref.			44	Ref.		
	≥ 70	231	0.12	−0.09; 0.33	0.253	187	0.12	−0.12; 0.35	0.332	44	−0.09	−0.53; 0.34	0.665
Gender	Males	341	Ref.										
	Females	88	0.24	−0.02; 0.51	0.072								
Former UCa	No	268	Ref.			208	Ref.			60	Ref.		
	Yes	161	−0.09	−0.32; 0.13	0.403	133	−0.19	−0.45; 0.07	0.151	28	0.19	−0.21; 0.60	0.346
Smoking Status	Never smoker	94	Ref.			51	Ref.			43	Ref.		
	Former smoker	220	−0.13	−0.39; 0.14	0.348	195	−0.06	−0.39; 0.28	0.740	25	−0.48	−0.95; −0.02	0.042
	Current smoker	115	0.09	−0.21; 0.39	0.545	95	0.12	−0.25; 0.50	0.514	20	−0.02	−0.54; 0.51	0.954
Leucocytes	Negative	239	Ref.			211	Ref.			28	Ref.		
	Positive	190	0.09	−0.13; 0.30	0.432	130	0.11	−0.14; 0.35	0.404	60	−0.03	−0.44; 0.39	0.891
Erythrocytes	Negative – ∼10 Ery/μL	226	Ref.			184	Ref.			42	Ref.		
	∼25 – 50 Ery/μL	76	0.55	0.27; 0.83	<0.001	55	0.58	0.25; 0.91	0.001	21	0.46	−0.01; 0.94	0.057
	∼150 – 250 Ery/μL	127	0.81	0.54; 1.07	<0.001		0.89	0.58; 1.19	<0.001	25	0.35	−0.16; 0.85	0.175

Persons with a complete data set (429 out of 484) were used, while those with missing values in one or more category were excluded; Parameter estimates are given as Δ with 95% confidence intervals (CI).

**Figure 2:**
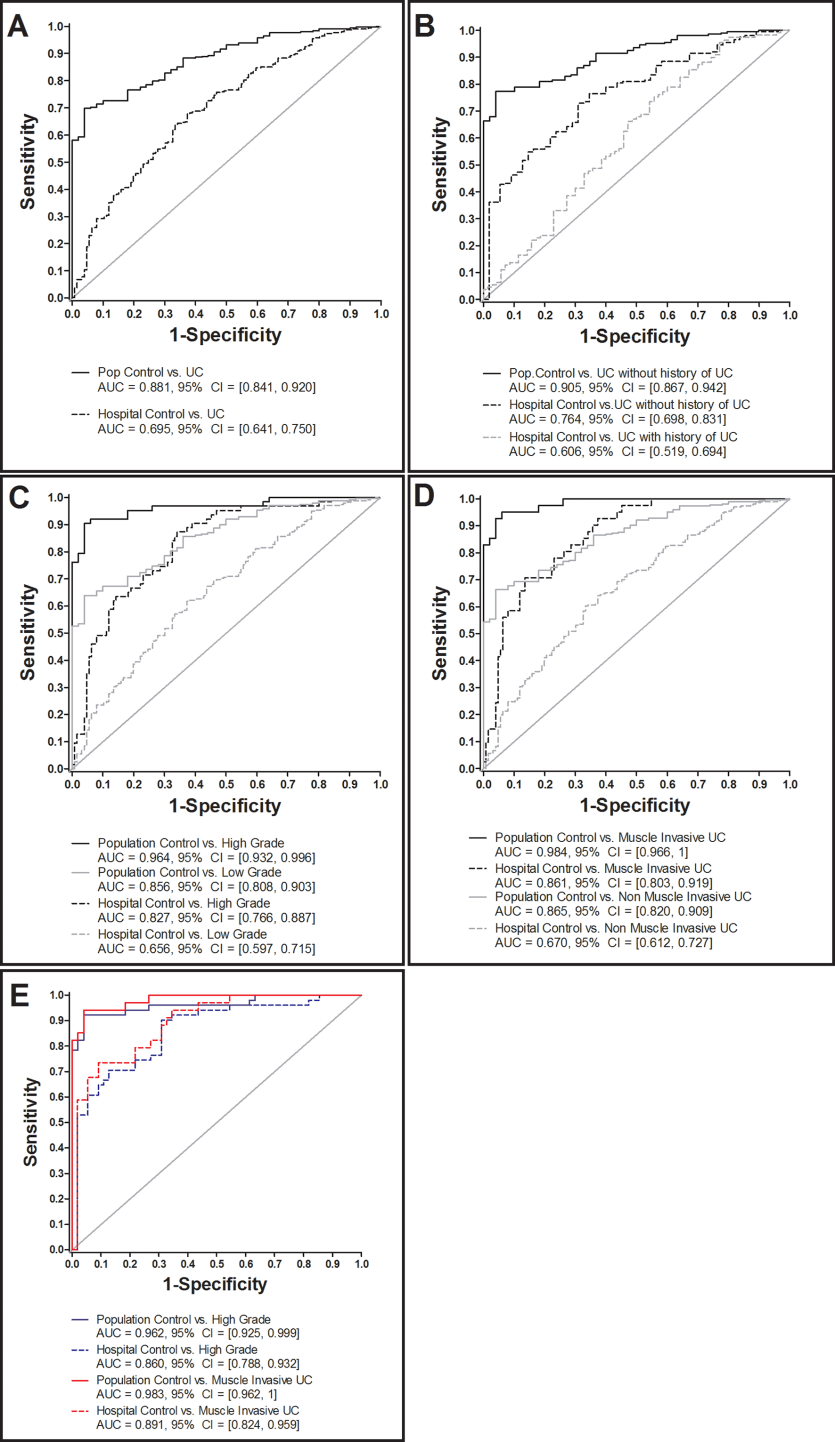
ROC analysis of creatinine-normalized CXCL16 in urine of urothelial carcinoma (UC) patients and various control subjects Comparison of UC patients with population and hospital controls **(A)**. UC patients with and without former UC history versus population and hospital controls **(B)**, high- and low-grade UC compared to population and hospital controls **(C)**, muscle invasive and non-muscle invasive UC versus population and hospital controls **(D)**, and high-grade or rather muscle-invasive UC without UC history versus population and hospital control in terms of a best-fit analysis **(E)**.

Receiving Operator Characteristics (ROCs) analysis showed increased sensitivity and specificity of CXCL16 towards UC, compared to population (AUC 0.88, 95% CI 0.84 – 0.92) and urological hospital controls (AUC 0.70, 95% CI 0.64 – 0.75) (Figure 2A). Separate ROC analysis of UC patients that distinguished between primary (=without history of UC) and recurrent UC (=with history of UC) resulted in higher AUC values in primary UC patients compared to population (AUC 0.91, 95% CI 0.87 – 0.94) and urological hospital controls (AUC 0.76, 95% CI 0.70 – 0.83) (Figure 2B). A lower AUC and 95 CI, however, were observed upon comparing recurrent UC and hospital controls (AUC 0.606, 95% CI 0.519 – 0.694). No comparison could be carried out between population controls vs. recurrent UC due to the fact that all population controls were without history of UC.

Urinary CXCL16 concentration was further evaluated for its ability to discriminate low grade from high grade UC patients and muscle invasive from non-muscle invasive UC, as this represents a relevant biomarker application with regard to treatment decision. ROC analysis stratified by grading and staging of UC patients versus the control groups showed a distinct increased sensitivity and specificity in high-grade compared to low grade UC patients (Figure 2C), and in muscle-invasive compared to non-muscle invasive UC patients (Figure 2D). In summary, the overall best discrimination results were observed for primary UC patients with high-grade and muscle-invasive UC versus control subjects (AUC ≥0.86, Figure 2E). In order to discriminate high grade and muscle-invasive UC cases from hospital controls and low grade UC cases (independent from UC history), the sensitivities and specificities of CXCL16 based on the 95^th^ percentile of CXCL16 in hospital controls (648.52 pg/mg creatinine) were calculated. Consequently, high grade UC vs. hospital controls revealed a sensitivity of 34.9% and a specificity of 94.4%, muscle-invasive UC a sensitivity of 41.5% and a specificity of 94.4%. Comparing high grade vs. low grade UC resulted in a sensitivity of 34.9% and specificity of 85.7%.

### CXCL16 and CXCR 6 in tissue

In order to investigate the expression of CXCL16 and its G-protein-coupled receptor CXCR6, immunohistochemical staining of paraffin-embedded tissues was initially performed in a subset of 17 UC patients (n=13 low grade, n=4 high grade) and 15 hospital controls, with histologically confirmed urocystitis only. Three different methods/criteria were used to evaluate the results, and all approaches revealed distinct staining differences between both groups (see Materials and Methods).

**Figure 3:**
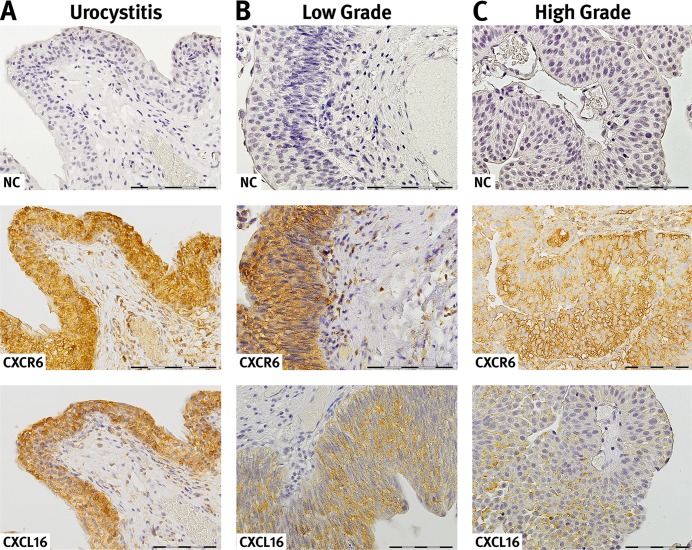
Expression and localization of CXCR6 and CXCL16 in urothelial tissue CXCR 6 and CXCL16 immunoreaction in acute urocystitis **(A)**, low grade **(B)** and high urothelial carcinoma **(C)**. CXCR 6 and CXCL16 are expressed on the surface as well as in the cytosol of epithelial cells. Moreover, in certain areas there is a strong co-localisation of the receptor-ligand pair observable. For detection of negative immunoreactivity (NC) within the same tissue section a consecutive section was stained. The presented slides reveal representative examples of the CXCL16 and CXCR6 immunoreactivity. Magnification, x40; bar 100 μm.

Eighty-seven percent of the urocystitis samples had an intermediate and strong staining for CXCL16 when the staining intensity was evaluated across the whole slide. Similar results (84.4%) were observed when randomly flagged areas of epithelium or cancer cells were evaluated within a pre-defined area within the stained area. In contrast, CXCL16 staining in UC cases was negative or weak in 94% (across slide evaluation) and 61.1% (random area evaluation). The results of both methods, although not completely identical, agree with each other and show a clear shift towards intermediate and strong staining of CXCL16 in urocystitis; whereas, CXCL16 staining in UC is mostly absent or weak. Finally, intermediate to strong staining of CXCL16 in urocystitis was quantified using an H-score and revealed staining intensity that was twice as high (238) compared to UC (138) (p=0.0016) (Table 3, Figure 3). These results were confirmed by additional CXCL16 stainings in tissue of 33 UC patients (n=14 low grade; n=22=high grade UC patients) and 16 patients with an inflammation in the bladder, resulting in a total sample set of 53 UC patients and 35 patients with urocystitis. The most prominent staining of CXCL16 in the urocystitis patients (hospital controls) was detected in epithelial cells and in tissue remodelled by inflammatory processes.

**Table 3:** Semiquantitative analysis of CXCR6 and CXCL16 immunoreactivity in tissue specimen of patients with primary urothelial cancer (UC; n=13 low grade, n=4 high grade) or an inflammation of the bladder (Uro)

Immunoreactivity	CXCL16		CXCR6	
Across the whole slide (Method 1)	UC (n=17)	Uro (n=15)	UC (n=17)	Uro (n=15)
Absent, n	**3 (18%)**	0 (0%)	0 (0 %)	0 (0%)
Weak, n	**13 (76%)**	2 (13%)	2 (12%)	0 (0%)
Intermediate, n	1 (6%)	**10 (67%)**	**7 (41%)**	**5 (33%)**
Strong, n	0 (0%)	**3 (20%)**	**8 (47%)**	**10 (67%)**
Percentage of stained area (Method 2)	**Mean**	**Mean**	**Mean**	**Mean**
Absent	**12.3%**	0%	0%	0.7%
Weak	**48.8%**	16.7%	0%	6.6%
Intermediate	27.7%	**28.5%**	**29.6%**	**10.5%**
Strong	11.1%	**55.9 %**	**70.4%**	**82.2%**
H-Scoring (Method 3)	**Mean ± SD**		**Mean ± SD**	
	137.65±72.92	237.93±62.59^*^	270.37±27.45	283.44±34.62

n, number of samples; Mean±standard deviation H-scores in UC and patients with an inflammation of the bladder; ^*^p=0.0016 p-value from Mann-Whitney test.

Compared to CXCL16, no differences were observed in the staining intensity of CXCR6 between UC and urocystitis patients. This was true for all three tested staining and evaluation criteria (Table 3). The immunoreactivity of the CXCR6 receptor was similar (intermediate and strong) in all cells from primary UC patients and hospital controls (H-Score: 270.4±27.5 in UC patients vs. 283.4±34.6 in patients with inflammation of the bladder). In UC specimens, CXCR6 expression was consistently intermediately or strongly expressed in tumour cells and infiltrating inflammatory cells. In specimens from hospital controls, CXCR6 immunostaining signal was particularly strong in epithelial cells and areas remodelled by inflammatory processes. CXCR6 was located both in the cytoplasm and cell membrane, and showed strong co-localization with its receptor, CXCR6 in cancer cells and epithelial cells. Conversely, CXCL16 was primarily found in the cell membrane and cytosol of cells. Since there were no differences in CXCR6 staining intensities between UC and urocystitis, no further follow up was carried out in the extended sample set of 53 UC and 35 urocystitis patients.

### CXCL16 in matched tissue and urine samples

**Figure 4:**
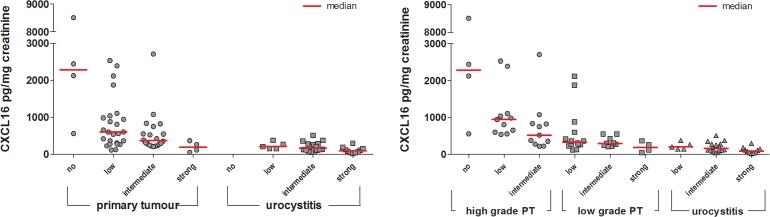
Semi-quantitative analysis of CXCL16 immunoreactivity in primary tumours (high and low grade) and urocystitis tissues in relation to the CXCL16 concentration found in the urine supernatant of these patients Staining of CXCL16 in patient tissue was categorized as absent, low, intermediate or strong according to method 1 (Table 3). Normalized CXCL16 concentrations in urine supernatant were compared to the immunoreactivity signal found in the matching tissues.

A comparison of urinary CXCL16 concentration with CXCL16 immunostaining in the same tissue sample revealed a negative correlation between tissue and urinary CXCL16. Indeed, the higher the CXCL16 staining in tissue, the lower the CXCL16 concentration in the corresponding patient's urine supernatant (Figure 4). Accordingly, urine supernatant from high grade UC patients had high urinary CXCL16 concentrations; whereas, CXCL16 staining of cancer cells was low in the corresponding tissue. In contrast, CXCL16 levels were intermediate to high in tissue samples from patients with low grade UC and urocystitis, while its levels in urine were low.

## DISCUSSION

In this cross-sectional study with UC cases and two groups of controls (urological hospital controls and population controls), we identified CXCL16 as a novel biomarker candidate for UC with a preference for high-grade, muscle-invasive and primary UC. CXCL16 was excreted in about 4-fold higher concentrations in pre-therapeutic urine samples from patients with high-grade UC compared to voided urine samples from hospital and population controls. ROC analyses resulted in AUC values of 0.83 and 0.96 for high-grade tumours compared to hospital and population controls, respectively. Sensitivity was 62% (at a pre-defined specificity of ≥95%) for high-grade UC versus hospital controls, and 94% in comparison to population controls. CXCL16 showed increased sensitivity and specificity for primary UC with AUC values of 0.76 and 0.91 compared to hospital and population controls upon analysis of the group of UC patients with regard to their individual UC history (primary vs. recurrent UC). This was expected because primary UC are usually larger in size and higher in stage and grade compared to recurrent UC and, therefore, can excrete higher levels of cancer-specific biomarkers. Accordingly, patients with muscle-invasive UC (>pT1) showed an increased specificity and sensitivity of CXCL16 compared to hospital controls with an AUC value of 0.86. Our study also suggests that CXCL16 is a promising high-grade UC marker, independent of UC history because AUC for high-grade UC in both primary and recurrent cancers was high (AUC 0.875 – 0.783) compared to the hospital controls (Figure 2D). Because this result is from a small subset of high-grade tumour samples (n=12 with history and n=50 without), further validation is required. In addition, the prevalence of high-grade tumours within primary UC cases was 35%, but only 12% for recurrent UC.

The good performance of CXCL16 supports it as a candidate for companion diagnostics, especially in order to verify high-grade UC. The latter, according to current WHO 2016 guidelines, requires a thorough and in-depth pathological evaluation of the complete tissue sample in order to identify even the smallest sections of high-grade lesions within a given tissue sample, ideally before penetrating the basal membrane. Therefore, the fast and reliable companion diagnostics for CXCL16 in urine by ELISA can be a useful tool in assisting pathologists when grading the tissue sample. This is particularly advantageous in those cases where the complete tissue sample either (1) cannot be completely screened, or (2) when a confirmation analysis for low-grade UC is needed. The latter is of particular importance to prevent misdiagnosis of a high grade tumour due to different therapeutic approaches when treating patients with high and low grade UC.

Based on our results and the high variability of CXCL16 concentrations in urine between subjects in both cases and controls, CXL16 is not proposed to be a stand-alone diagnostic screening marker for UC (general diagnostics) in asymptomatic persons, e.g., the general population. Nevertheless, it may prove worthwhile to include CXCL16 (or other high grade biomarkers) in future studies as part of a protein biomarker panel.

When we explored potential modifying factors of CXCL16 using a multiple linear regression model and stratifying according to both individual characteristics of the subjects (e.g., age, gender) and clinically relevant characteristics of the patients’ urine samples (e.g., leucocyte and erythrocyte counts), we found that gender, smoking status and age did not influence CXCL16. In our patient group, we also did not observe a significant influence of leukocytes in urine on the concentration of CXCL16. In contrast, leucocytes are well-known and important confounders of NMP22 and CFHrp [10, 11], two FDA-established protein biomarkers in urine that are currently used in clinical practice. However, CXCL16 levels appeared to increase with the presence of erythrocytes. Therefore, the ability to reliably diagnose high grade UC in the presence of urinary erythrocytes needs to be further investigated in more detail, and separated cut-offs of CXCL16 (with and without erythrocytes in urine) may have to be established.

The limitations of our cross-sectional design in marker identification studies have been addressed in many reviews [14, 15]. As a consequence, we lack pre-diagnostic samples from cases and may therefore overestimate the CXCL16 concentrations in cases that already have clinical symptoms due to a larger tumour. Conversely, as already mentioned above, CXCL16 is only suggested for companion diagnostics rather than general diagnostics in asymptomatic individuals.

A major strength of our study is the use of two different control groups: urological hospital controls and population-based control subjects. Urological hospital controls frequently suffer from conditions, such as urinary tract infections, which are known to affect protein-based markers, i.e., NMP22 [13]. Therefore, the comparison of marker levels between UC patients with urological hospital controls was an important part of our analysis, rather than using population-based controls only. In addition, the main source population (urological hospital controls and UC patients) and recruitment procedures very closely represented typical clinical and residence urologists’ situations. In such settings, high-risk patients (e.g. with haematuria at primary diagnosis, with abnormal cystoscopy results or under surveillance) are most likely to benefit from the adoption of a non-invasive urine test for diagnosis. Nevertheless, separate studies testing CXCL16 against control patients who reflect the range of different pathologies that are found in daily urologic or gynaecologic practice, such as (micro)haematuria, infection, urolithiasis, and urological and gynaecological cancers (other than UC) would be an important further analysis. Because our results specifically highlight the use of CXCL16 for companion diagnostics with a preference for primary and high grade UC, a highly specific validation in such a collective is of particular importance. The marker may be most relevant for patients with high grade T1 disease, as they are believed to be understated and develop recurrences.

Immunohistochemistry analysis showed that the increased excretion of CXCL16 in urine was accompanied by a decreased expression in the corresponding tissue samples of UC patients; whereas, receptor expression (CXCR6) remained unaffected between cancerous and normal urothelium. CXCL16 was first identified in early 2000 as an important player in immune defence, i.e., attracting natural killer T cells [16]. Therefore, maintaining steady-state levels of CXCL16 in tissue is thought to be beneficial for the recognition and elimination of diseased cells. Although no specific data are available on CXCL16 and bladder cancer, our results are in line with those reported in renal cancer, where CXCL16 correlated inversely with tumour stage, i.e. high tissue levels of CXCL16 was associated with lower stage and better patient survival [17]. In addition, normal renal tissue showed high endogenous CXCL16 expression, which in turn suggests that reduced CXCL16 expression is linked to cancer development in this organ [18]. This assumption is further supported by other studies investigating CXCL16 in solid tumours, including non-small cell lung cancer [19] and colon cancer [20, 21]. Accordingly, a recent study described high CXCL16 expression in lung cancer cells as a positive prognostic factor [22].

CXCL16 exists in two forms: a transmembraneous (tm) and a soluble (s) form [23]. Several studies have shown that sCXCL16 induce migration and proliferation of cancer cells; whereas, tmCXCL16 inhibits cell growth [23]. Our results suggest that UC cells increase the secretion of sCXCL16 in urine in order to prevent adhesion of immune cells via tmCXCL16, and, therefore, UC cells might avoid recognition and clearance by the host immune system. For example, the disintegrin and metalloproteinase 10 (ADAM 10) has been shown to regulate the cleavage and shedding of CXCL16 [24], and it was also demonstrated that ADAM 10 regulates the proliferation, invasion and chemoresistance of bladder cancer cells [25]. Escaping immune defence by altering chemokine homeostasis is one of the major hallmarks of cancer, and is often associated with progression, invasion and metastasis [26]. Therefore, CXCL16 may be an interesting candidate marker for follow-up studies to assess its predictive value for cancer recurrence and progression. Our results further support this function because sCXCL16 was predominantly found in the urine of patients with high grade and muscle invasive tumours. In addition, recently published data also suggest that advanced UC with low prognosis can be specifically targeted with immune therapy [27, 28]. Altogether, both previously published results, as well as our findings support a vital role of escaping immune defence in advanced UC.

In summary, we present CXCL16 as a promising biomarker candidate for high-grade or muscle invasive UC that is suitable for companion diagnostics in UC patients. Our results on CXCL16 in UC tissue, together with those from previously published data in other solid tumours suggest a critical role for this chemokine in tumour progression, patient prognosis, and as a potentially successful treatment by targeted immunotherapy.

## MATERIALS AND METHODS

### Study design and urine sample preparation

A cross-sectional study design using three different groups of persons (308 UC patients, 126 patients with histologically confirmed urocystitis but without UC, 50 population controls) was used to identify and verify potential non-invasive biomarkers for UC in urine. Two phases, a semi-quantitative screening approach using a commercially available antibody assay and a quantitative approach using ELISA, were designed. All spot urine samples were collected in the morning, centrifuged, and stored at −80°C. In the case of patients (UC and urocystitis), all urine samples have been collected before cystoscopy, upper urinary tract inspection, and treatment by transurethral resection (TURBT). In case of muscle invasive tumors no additional (second) urine sample was collected later on, e.g., directly before cystectomy. Creatinine was determined according to Jaffé [27]. In the hospital samples and in the population controls, the presence of leukocytes (yes: >10 leukocytes/μl urine vs. no) and number of erythrocytes (in categories: negative-∼10, ∼25-50, ∼150-250 erythrocytes/μl urine) in urine were performed with Combur-Test^®^ sticks (Roche). The study was approved by the Ethics Committee of the Ruhr-University Bochum (no. 3674-10), and all participants provided written informed consent.

### Antibody array

The initial screen for potential biomarker candidates was carried out using a commercially available antibody array capable of simultaneously screening the relative levels of 55 angiogenesis-related proteins (R&D Systems). For this purpose, we used a targeted approach of analysing a limited number of highly selected (matched) urine samples. Urine samples from six primary UC patients (all low grade, without history of UC) and six hospital controls with pathologically confirmed urocystitis were used. Samples were drawn from the bladder cancer biobank of the PURE consortium (Protein Research Unit Ruhr within Europe) at IPA and specifically matched for gender (4 males and 2 females in each group), smoking status (1 smoker, 5 non-smokers in each group) and age (median age 72 years in each group). All samples were free of potential confounders such as leucocytes and erythrocytes, a prerequisite for initial screening purposes. The protocol of the antibody array, originally designed by the manufacturer for investigating sera or saliva, was adapted for its use to examine urine samples. In short, membranes immobilized with the 55 different capture antibodies printed in duplicate were blocked for 1h at RT. In parallel, samples (500 μl untreated urine supernatant/membrane) were incubated with biotinylated Detection Antibody Cocktail for 1h at RT. Membranes were then incubated with the samples overnight at 6°C on a rocking platform. After extensive washing using 20 ml of 1x Wash Buffer (3x times, 10 min), membranes were incubated with horseradish-peroxidase-conjugated streptavidin for 30 min at RT. After washing 1x Wash Buffer (3x times, 10 min), the spot signals were detected by enhanced chemiluminescence (Pierce ECL, Thermo Fisher Scientific, Bonn, Germany). The array image was analysed using the LabImage 1D software (Kapelan, Leipzig, Germany). For this purpose, the pixel density in each spot was analysed. The average signal of a pair of duplicate spots representing each protein was determined and background corrected before analysis. Finally, corresponding signals on two arrays (urothelial carcinoma *vs*. urocystitis) were compared to semi-quantitatively determine the relative change of protein levels between samples.

### Enzyme-linked immunosorbent assay

For quantification of CXCL16 in urine we used the custom human CXCL16 ELISA Kit (RayBiotech) as described by the manufacturer. All samples (urine supernatant) were measured in duplicate and confirmed in two independent experiments. Creatinine-standardized CXCL16 concentrations (pg/mg) were calculated to normalize the results [29].

### Immunohistochemistry

Immunohistochemical staining of paraffin-embedded tissues was performed as previously described [30]. Paraffine-embedded tissues from urothelial cancer or acute urocystitis were cut in 1-μm sections and then used for staining. In brief, after drying overnight at 37°C, sections were deparaffinised in Rotihistol and subsequently hydrated through graded alcohol series. Antigen retrieval was performed in Target Retrieval Solution pH 6 for 20 min (Dako). Sections were then blocked for unspecific protein binding with T-PBS, and for endogeneous peroxidase by use of Dako Dual Endogenous Enzyme Block. The following primary antibodies were used: CXCL16 (1:250; Acris Antibodies) or CXCR6 (1:750; Abcam). As negative control, sections were incubated without using a primary antibody (Dako). After incubation overnight at 4°C, the antigen was stained brown by the use of the Dako Envision-Kit according to the manufacturer`s protocol. For a blue nuclear counterstaining, specimens were incubated with haematoxylin (2 min, Dako). Finally, samples went through a series of ascending alcohol concentrations and were mounted with Entellan (Merck, Darmstadt, Germany). For semi-quantitative analysis, the stained slides were scanned at 20x magnification using the Nanozoomer whole slide scanner from Hamamatsu (Herrsching am Ammersee, Germany); the images were then evaluated by using the viewer software NDP.view2 (Hamamatsu). For semi-quantitative analysis, slides were scored independently by two observers for quality control as described previously [31]. In a first run with 32 samples in total (n=17 primary UC and n=15 urocystitis) staining intensity of CXCL16 and CXCR6 in epithelium or cancer cells across the whole slide was categorized as absent, weak, intermediate or strong. In the case of divergent scoring, a third observer decided upon the final category. In a second run, we analysed the same slides, but flagged randomly selected areas of epithelium or cancer cells with a pre-defined area of 0.6 mm^2^ in patients with an inflammation of the bladder and 2.0 mm2 in patients with UC using the annotation tool of NDP.view2. These areas were, if necessary, further subdivided in smaller areas and all selected areas were then categorized according to their staining intensities as absent, weak, intermediate or strong. At the end, we calculated the percentage of every staining category with regard to the pre-defined area. In a third run, we further analysed the results orientating at the H-score system (ranging from 0-300) [32]. Therefore, the intensity of the stain was multiplied by the percentage (0-100) of area showing that staining intensity: [1 x (% area with weak staining) + 2 x (% area with intermediate staining) + 3 x (% area with strong staining)]. Subsequently, we performed additional CXCL16 stainings in tissue of 33 UC patients (n=14 low grade; n=22=high grade UC patients) and 16 patients with an inflammation in the bladder (based on an across-read of the slides identical to the first run because we obtained comparable results for all three methods, see section Results).

### Statistical analysis

The distribution of CXCL16 values is presented by median and inter-quartile range (IQR). Group comparisons were conducted with non-parametric Wilcoxon rank-sum tests and graphically displayed with box plots. The potential influencing factors age (<70 years, ≥70 years), gender (male, female), smoking status (never, former, and current smoker), presence of urinary leukocytes (>10 leukocytes/μl vs. negative) and erythrocytes (>10 erythrocytes vs. negative) were evaluated with a multiple linear regression model after log-transforming the CXCL16 values in order to achieve a better approximation to the normal distribution. Subjects with missing values in one or more of the analysed variables were not included in the model. Parameter estimates were given as Δ with 95% confidence intervals (95% CI). Values of Δ>0 indicated an increase in CXCL16 levels compared to the reference, values of Δ<0 a decrease. Receiver operating characteristic curves (ROC) for CXCL16 were constructed and the area under curve (AUC) was determined. All calculations were done using SAS (version 9.4). P-values <0.05 were considered as statistically significant.
